# Lessons from the ACDC-RP trial: Clinical trial design for radical prostatectomy neoadjuvant therapy trials

**DOI:** 10.18632/oncotarget.28648

**Published:** 2024-09-30

**Authors:** Rashid K. Sayyid, Neil E. Fleshner

**Keywords:** clinical trial, prostatic neoplasms, neoadjuvant therapy, chemotherapy, androgen receptor agonist

To date, clinical trials of neoadjuvant therapy have failed to demonstrate significant survival benefits in prostate cancer patients undergoing a radical prostatectomy [1]. Clinical trials in this space have been limited by their inclusion of lower risk patients, small sample size, short-term follow-up, reliance on pathologic outcomes as primary study endpoints, and duration/choice of neoadjuvant systemic therapy [2–7].

ACDC-RP (NCT02543255) was an open-label, multicenter, phase II trial that randomized 70 men with clinically localized, D’Amico high-risk prostate cancer to chemohormonal therapy with neoadjuvant cabazitaxel (25 mg/m^2^) plus abiraterone acetate (1,000 mg/day) and leuprolide acetate versus abiraterone acetate plus leuprolide acetate. It was hypothesized that the addition of cabazitaxel would help target androgen-insensitive clones, such as those with *PTEN* mutations. This trial failed to meet its primary endpoint of improved pathologic complete response or minimal residual disease with the addition of cabazitaxel (43.2% and 45.5% in experimental and control arms, respectively). Pathologic complete responses were observed in two (5.4%) and three (9.1%) patients in the experimental and control arms, respectively. Patients with a pathologic complete response or minimal residual disease had superior 12-month biochemical recurrence-free survival rates (96% versus 62%, *p* = 0.03), confirming the clinical relevance of the primary study endpoint. Grade ≥3 adverse events were observed in 42.5% and 23.7% of patients in the experimental and control arms, respectively (*p* = 0.078) [8].

While the ACDC-RP trial adds to the litany of negative trials in this disease space, there are key takeaways from this trial that can inform future clinical trial design. Almost half of the patients in both the experimental and control arms achieved a pathologic complete response or minimal residual disease on the radical prostatectomy specimens. These results are consistent with those observed in the recently published ARNEO trial of neoadjuvant apalutamide (38%) [9] and the 2014 trial by Taplin et al. of neoadjuvant abiraterone acetate (62%) [10] and considerably higher than those observed in historic trials of neoadjuvant luteinizing hormone-releasing hormone (LHRH) agonists and 1st generation anti-androgens [3–7]. We believe that these results highlight the importance of maximal androgen blockade with an androgen receptor pathway inhibitor in the neoadjuvant setting and should serve as the ‘backbone therapy’ for clinical trial neoadjuvant regimens for high-risk prostate cancer patients. There are numerous ongoing trials evaluating androgen receptor pathway inhibitors in the neoadjuvant setting, including the phase 3 PROTEUS trial (NCT03767244) of perioperative androgen deprivation therapy (ADT) plus either apalutamide or placebo, administered for 6 months both neoadjuvantly and adjuvantly [11].

How do we build upon trials of neoadjuvant androgen receptor pathway inhibitors for high-risk prostate cancer? One such approach may be adopting a biomarker-selected treatment algorithm. It has been demonstrated that approximately 6% of clinically localized, high-risk prostate cancer patients harbor pathogenic germline mutations, with germline-only testing (i.e., no somatic testing) missing nearly half of all homologous recombination repair mutations [12, 13]. NePtune (NCT05498272) is a single arm phase II trial evaluating the neoadjuvant combination of olaparib, a Poly (adenosine diphosphate-ribose) polymerase (PARP) inhibitor, plus an LHRH agonist for six months followed by radical prostatectomy in clinically localized, high-risk prostate cancer patients with germline or somatic *BRCA1/2* alterations. The primary study endpoint is pathologic complete response or minimum residual disease, as determined by central pathology review [14].

The Genomic Biomarker-Selected Umbrella Neoadjuvant Study for High Risk Localized Prostate Cancer (GUNS) trial (NCT04812366) trial is an adaptive, multi-arm, multi-stage trial designed to evaluate targeted therapies in biomarker-pre-selected patients with high-risk localized disease by matching neoadjuvant therapies to baseline genomic alterations. All enrolled patients will initially receive eight weeks of apalutamide plus an LHRH analogue while genomic profiling and trial arm assignment is ongoing. Patients will be assigned into one of four groups based on their genomic alteration status:

- Group 1 will include patients without any androgen receptor-axis targetable alterations. They will be randomized to an LHRH analogue + apalutamide +/− abiraterone acetate.
- Group 2 will include patients with loss of tumor suppressor gene alterations (e.g., *PTEN, RB, p53).* They will be randomized to receive an LHRH analogue + abiraterone acetate +/− docetaxel.
- Group 3 will include patients with DNA damage response alterations (e.g., *BRCA1/2)*. All patients in this group will receive an LHRH analogue + abiraterone acetate + niraparib.
- Group 4 will include patients with hypermutations, microsatellite instability, Lynch syndrome or CDK12 mutations. These patients will receive an LHRH analogue + apalutamide + atezolizumab, a Programmed Death-Ligand 1inhibitor.

In summary, recent evidence from clinical trials of neoadjuvant therapy prior to radical prostatectomy strongly suggest that maximal androgen blockade with an androgen receptor pathway inhibitor + an LHRH analogue should serve as the ‘backbone’ of neoadjuvant therapy trials. We await the results of ongoing trials evaluating biomarker-selected approaches in this disease space.
